# Immune Control, HBsAg Loss or Flare Requiring Liver Transplant—Diverse Outcomes After Stopping NUC Therapy

**DOI:** 10.3390/v18091026

**Published:** 2026-09-15

**Authors:** Katarina Lund, Miriam Frankal, Anders Eilard, Joakim Bedner Stenbäck, Erik Alestig, Arno Furquim d’Almeida, Staffan Nilsson, Thomas Vanwolleghem, Johan Westin, Johan Ringlander, Gustaf E. Rydell, Magnus Lindh

**Affiliations:** 1 Department of Infectious Diseases, Norra Älvsborg’s Hospital, 461 73 Trollhättan, Sweden; 2 Department of Infectious Diseases, Institute of Biomedicine, Sahlgrenska Academy, University of Gothenburg, 405 30 Gothenburg, Sweden; 3 Department of Infectious Diseases, Södra Älvsborg’s Hospital, 501 82 Borås, Sweden; 4 Department of Infectious Diseases, Sahlgrenska University Hospital, 413 46 Gothenburg, Sweden; 5 Viral Hepatitis Research Group, Laboratory of Experimental Medicine and Pediatrics, University of Antwerp, 2610 Antwerp, Belgium; 6 Department of Gastroenterology and Hepatology, Antwerp University Hospital, 2650 Antwerp, Belgium; 7 Department of Laboratory Medicine, Institute of Biomedicine, University of Gothenburg, 413 45 Gothenburg, Sweden

**Keywords:** hepatitis B virus, chronic hepatitis B, HBsAg, HBV DNA, liver transplantation, prospective study, follow-up

## Abstract

Hepatitis B surface antigen (HBsAg) loss is rarely achieved during long-term nucleos(t)ide analogue (NUC) therapy of chronic hepatitis B. Treatment discontinuation may lead to immune activation and HBsAg loss. In a prospective study, HBeAg-negative patients without liver cirrhosis stopped NUC therapy after a duration of at least 3 years. Follow-up for 2 years comprised monthly monitoring for the first six months. Twenty-five patients who discontinued therapy and six randomized controls who continued were included. After stopping NUC therapy, five patients (20%) developed an HBsAg seroclearance pattern, including four who lost HBsAg and one who achieved HBsAg below 1 IU/mL. Seven patients (28%) developed a state of immune control with persistently low HBV DNA and normal ALT. Six patients (24%) experienced severe flares requiring NUC re-initiation; all of them, including one with fulminant hepatitis necessitating liver transplantation, had detectable hepatitis B core-related antigen (HBcrAg) in serum at NUC discontinuation. The peak serum HBV DNA level strongly correlated with and preceded peak ALT. Thus, although half of the patients had favorable outcomes, there was a substantial risk of severe flares preceded by HBV DNA levels above 6 log_10_ IU/mL. Close monitoring, including frequent HBV DNA testing with rapid turnaround time, is therefore essential.

## 1. Introduction

Chronic hepatitis B (CHB) constitutes a major cause of liver cirrhosis and hepatocellular carcinoma (HCC). Long-term nucleos(t)ide analogue (NUC) therapy of CHB reduces the risk of these complications [1] but rarely achieves loss of HBsAg (‘functional cure’) [2], which is a key therapeutic goal [3]. Discontinuation of NUC therapy increases the likelihood of HBsAg loss in patients with HBeAg-negative CHB [4,5,6,7,8,9,10,11,12], but confers a risk of severe flare [12,13,14,15]. Selection of patients suitable for treatment discontinuation is therefore important and requires balancing the likelihood of a beneficial outcome against the risk for severe reactivation.

Low HBsAg serum levels at the time of discontinuation, below 1000 IU/mL or preferably below 100 IU/mL, are associated with a higher probability of HBsAg loss [6,7,11,12].

Other proposed predictors, including HBV genotype, levels of anti-HBc IgG or detection of hepatitis B core-related antigen (HBcrAg) or HBV RNA, may help identify patients at lower risk of severe flare [12,13,14,15,16,17], but these observations and proposed thresholds require further validation.

This prospective study investigated outcomes and predictors after discontinuation of long-term NUC therapy in patients with HBeAg-negative CHB who had received treatment for a median of 10 years. Approximately half of the patients achieved favorable outcomes, but 24% experienced severe flares, including one case requiring liver transplantation.

## 2. Materials and Methods

### 2.1. Patients

This investigator-initiated prospective clinical trial enrolled participants at infectious diseases clinics at three hospitals in Western Sweden between December 2022 and December 2024. Eligible patients had been HBeAg-negative at treatment initiation and had received NUC for at least three years. Exclusion criteria were age below 18 years, concomitant infection with hepatitis C virus, hepatitis D virus or HIV, or liver cirrhosis, assessed clinically or by liver stiffness > 12 kPa by FibroScan at pretreatment or at inclusion. Patients were randomized in a 3:1 ratio to discontinue or continue NUC therapy. Because recruitment was substantially lower than planned and only six control patients remained under follow-up, the control arm was used only as a descriptive reference for biomarker stability during continued therapy. A CONSORT flow diagram and checklist are provided as Supplementary Figure S1 and Table S1. Trial registration: ClinicalTrials.gov, identifier: NCT05328427; registered on 25 February 2022.

### 2.2. Aims

The predefined primary aims of the study were to determine the proportions of patients who, after discontinuation of NUC therapy, (i) achieved HBsAg seroclearance, (ii) developed a low-active state without further need for NUC, (iii) restarted NUC therapy because of insufficient control of viral replication, and (iv) experienced severe reactivation requiring NUC re-initiation. A secondary aim was to identify baseline predictors associated with these outcomes.

### 2.3. Monitoring and Laboratory Testing

Serum samples were obtained at NUC discontinuation, monthly until 6 months after stopping NUC, then every third month until 1 year after stopping NUC, and every 6 months thereafter. This report presents outcomes after two years. Analyses included alanine aminotransferase (ALT), platelets, PK-INR, albumin, bilirubin, HBV DNA, and quantitative HBsAg every 24 weeks. Additional samples were required within a week if HBV DNA exceeded 6.0 log_10_ IU/mL or when ALT/ULN (upper limit of normal) exceeded 10. Anti-HBc IgG and HBcrAg at discontinuation, and additional HBsAg quantifications were performed retrospectively.

### 2.4. Virological Analyses

HBV DNA was analyzed using the Cobas 6800 platform (Roche Diagnostics, Pleasanton, CA, USA; lower limit of quantification (LOQ) 1.0 log_10_ IU/mL), quantitative HBsAg using the Abbott Alinity assay (lower LOQ 0.05 IU/mL), HBcrAg (LOD 2 log_10_ U/mL; LOQ 3 log_10_ U/mL) and hepatitis B core immunoglobulin G antibodies (anti-HBc IgG) (LOD 0.5 IU/mL; LOQ 0.8 IU/mL) were analyzed on the Lumipulse G600II (Fujirebio, Tokyo, Japan). HBV genotyping was performed by whole-genome sequencing [18].

### 2.5. Criteria for Restarting NUC

Criteria to restart NUC were either of the following:

(i) Severe flare: repetitive HBV DNA > 6.0 log_10_ IU/mL or ALT > 10 × ULN with ≈ 1 week interval.
(ii) Unsatisfactory long-term outcome at the discretion of the treating physician guided by test results showing persistent HBV DNA > 20,000 IU/mL in combination with ALT > ULN, or persistent HBV DNA > 2000 IU/mL in combination with ALT > 2 × ULN.

### 2.6. Statistics

Potential associations between outcomes (HBsAg seroclearance, yes/no; immune control, yes/no; severe flare, yes/no) and age or level of HBsAg, HBcrAg, or anti-HBc IgG at NUC discontinuation were analyzed using the Mann–Whitney U test. Correlations were evaluated by linear regression analysis.

### 2.7. Ethics

Written informed consent was obtained from all subjects involved in the study. The study protocol complied with the 1975 Declaration of Helsinki and was approved on 3 December 2019 by the Swedish National Ethical Review Authority (Dnr 2019-04563).

## 3. Results

### 3.1. Participant Characterization

Thirty-five patients were included; 27 stopped NUC therapy, and eight were randomized to continue therapy as controls. Two controls withdrew consent soon after inclusion, leaving six patients in the control group. Two patients were found to have HBV DNA above 2 log IU/mL in samples taken at NUC discontinuation (due to poor adherence in one and lamivudine resistance in the other) and were excluded from the analyses. Baseline characteristics of the participants are shown in Table 1. All patients were HBeAg-negative, and none had liver cirrhosis. Median NUC therapy duration was 10 years in both groups. Two patients had received entecavir, and the remaining tenofovir disoproxil fumarate.

Among patients who discontinued NUC, median HBV DNA level prior to initiation of NUC therapy was 5.94 log_10_ IU/mL. At treatment cessation, the median HBsAg level was 3.08 log_10_ IU/mL, and HBcrAg was detected in 17 patients (68%), including 12 with quantifiable levels (≥3 log_10_ U/mL). Genotype D was predominant; nine patients had genotype B or C.

In patients who stopped treatment with tenofovir, viral reactivations occurred early and typically peaked at week 8 or 12. In the two who stopped entecavir treatment (patients i10 with insufficient control and s1 with borderline control), viral reactivations were slow and peaked at week 16 and 24, respectively.

### 3.2. Outcome Classification and Frequencies

Table 2 describes characteristics of the outcomes after discontinued therapy. Five patients presented an HBsAg seroclearance pattern (20%), defined as HBsAg decline to below 1 IU/mL, 12–96 weeks after discontinuation, including three with spontaneous HBsAg loss, one with spontaneous decline to 0.9 IU/mL after two years, and one who cleared HBsAg after restarting therapy (patient n3). Seven patients (28%) developed a state with HBV DNA persistently below 3.3 log_10_ IU/mL and normal ALT level, without indication for retreatment, here termed immune control. Eleven patients (44%) required re-initiation of NUC therapy: six due to severe flare and five due to insufficient immune control of viral replication. The remaining three patients did not fit into any of the four predefined outcomes; they had persistently normal ALT but HBV DNA > 3.3 log_10_ IU/mL and were classified as having borderline control.

Patients with the spontaneous HBsAg seroclearance pattern were older than the other patients (median 62.2 vs. 48.5; *p* = 0.016) and had received NUC therapy for a longer period (median 14 vs. 10 years; *p* = 0.032). The outcome groups did not differ in liver function (levels of platelets or albumin) or in liver stiffness at the time of NUC discontinuation.

### 3.3. HBsAg Seroclearance Pattern

**Figure 1:**
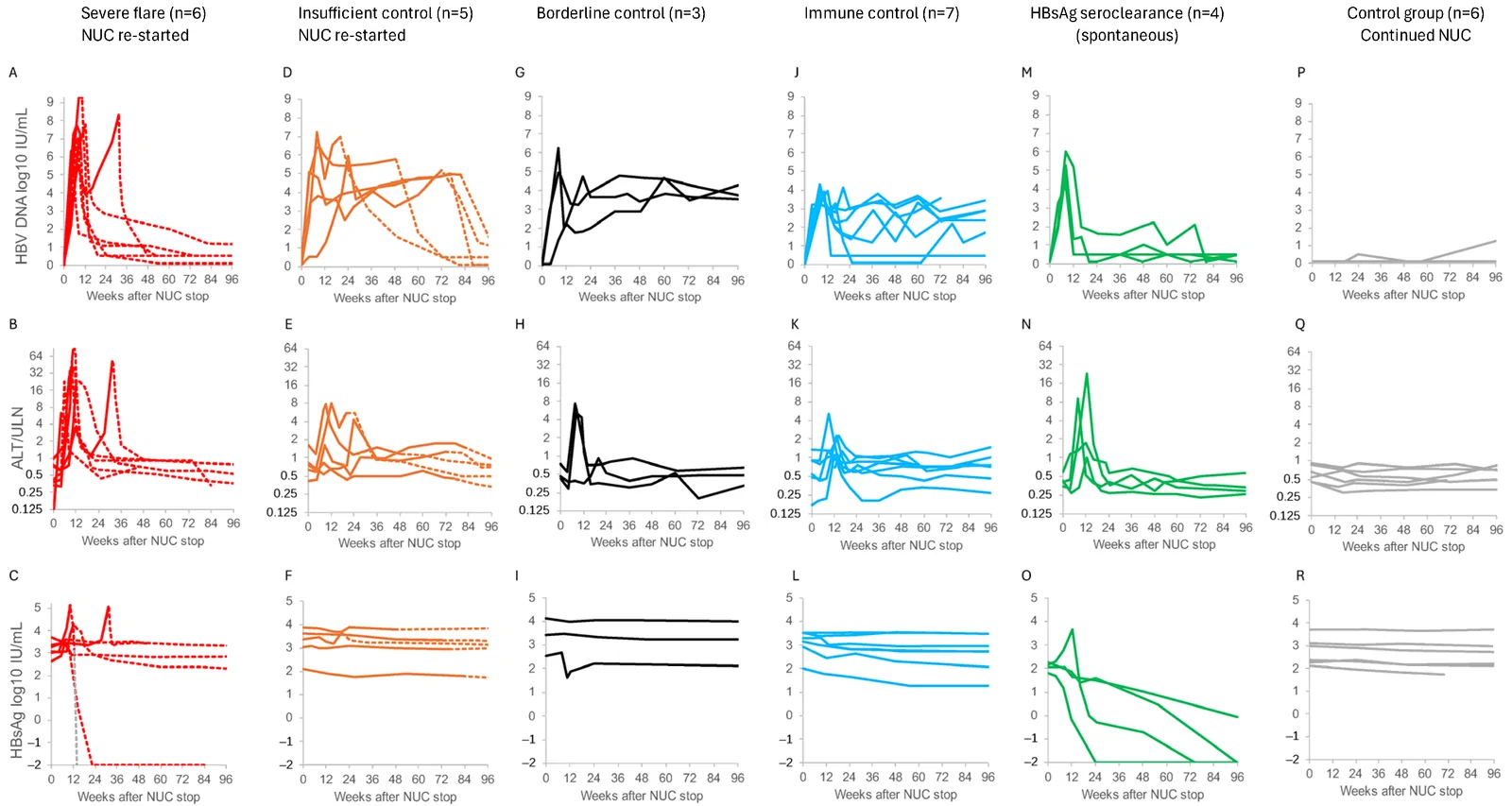
Changes over time of serum levels of HBV DNA (**upper**), ALT (**middle**) and HBsAg (**lower**) after stopping NUC therapy in patients classified into the outcome groups Severe flare (**A**–**C**), Insufficient control (**D**–**F**), Borderline control (**G**–**I**), Immune control (**J**–**L**), HBsAg seroclearance (spontaneous) (**M**–**O**) and control group (**P**–**R**). In plots (**A**,**B**,**G**,**H**), the dashed lines represent levels after re-initiation of NUC therapy. The grey dashed line in (**C**) shows the rapid HBsAg decline after liver transplantation in one case.

**Figure 2:**
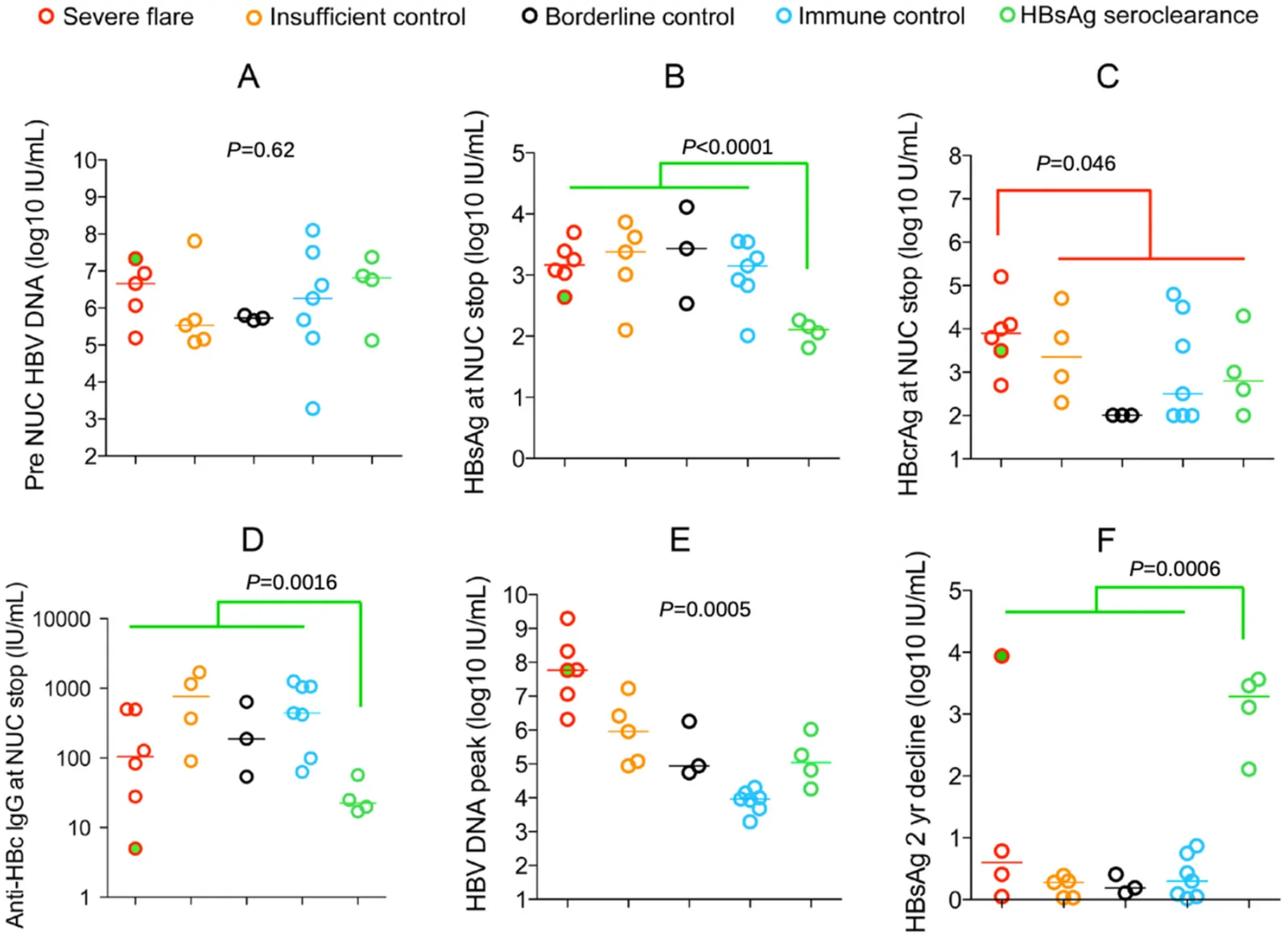
Virological markers at four time points: Before NUC therapy (**A**), at NUC discontinuation (**B**–**D**), at the peak of HBV DNA (**E**) and 2 years after NUC discontinuation (**F**). A green filling was added to the data point for the patient with severe flare who cleared HBsAg. Samples with undetected HBcrAg are shown as 2 log U/mL (LOD). One sample with undetected anti-HBc IgG is shown as 5 IU/mL. In the calculation of HBsAg log_10_ IU declines, samples with undetected HBsAg were given the value –1.3 log IU/mL (LOQ). Pre-NUC HBV DNA was missing for one patient. HBsAg decline at 2 years was missing in one patient with severe flare and is not shown for the liver transplant patient.

As described above, four patients (20%) presented a spontaneous HBsAg seroclearance outcome pattern (defined as reduction to below 1 IU/mL; Figure 1M–O; Supplementary Figure S2) and one cleared HBsAg after NUC re-initiation (Figure 1C; Supplementary Figure S6). As shown in Figure 2 and Supplementary Table S2, these patients had lower HBsAg levels at treatment discontinuation than others (median 2.11 vs. 3.25; *p* = 0.007 for spontaneous seroclearance). All five experienced distinct viral reactivations with HBV DNA peaks around 8 weeks after treatment cessation. Four had peaks between 4.26 and 6.02 log_10_ IU/mL, while the restarted patient reached 7.76 log_10_ IU/mL. Two had minor biochemical flares, two had ALT peaks at 9 and 23 × ULN, and the patient restarting NUC 40 × ULN. The patients with mild flares showed slow HBsAg declines; the other three showed rapid HBsAg clearance.

### 3.4. Immune Control

Immune control without HBsAg seroclearance occurred in seven patients (28%). This outcome was characterized by limited viral reactivations with a median peak HBV DNA level of 3.96 log_10_ IU/mL (range 3.29–4.31), mild biochemical flares with a median peak ALT at 1.27 × ULN (range 1.09–5.09). During follow-up, the HBV DNA levels remained below 3.3 log_10_ IU/mL and ALT was normal. Two showed a small reduction in HBsAg (Figure 1J–L; Supplementary Figure S3). Compared with prior to starting NUC therapy, their HBV DNA levels had decreased by a median of 4.60 log_10_ IU/mL (Supplementary Figure S8). This outcome could not be predicted by any of HBsAg, HBcrAg or anti-HBc IgG at the time of NUC discontinuation.

### 3.5. Severe Hepatic Flares

Six patients (24%) developed severe flares. Median HBV DNA peak was 7.77 (range 6.32–9.30) log_10_ IU/mL. In four patients, HBV DNA peaked 7–12 weeks after cessation; one peaked at week 4 and one at week 31. ALT peaked at levels above 20 × ULN at or within a few weeks after the viral peak (Supplementary Figure S6).

**Figure 3:**
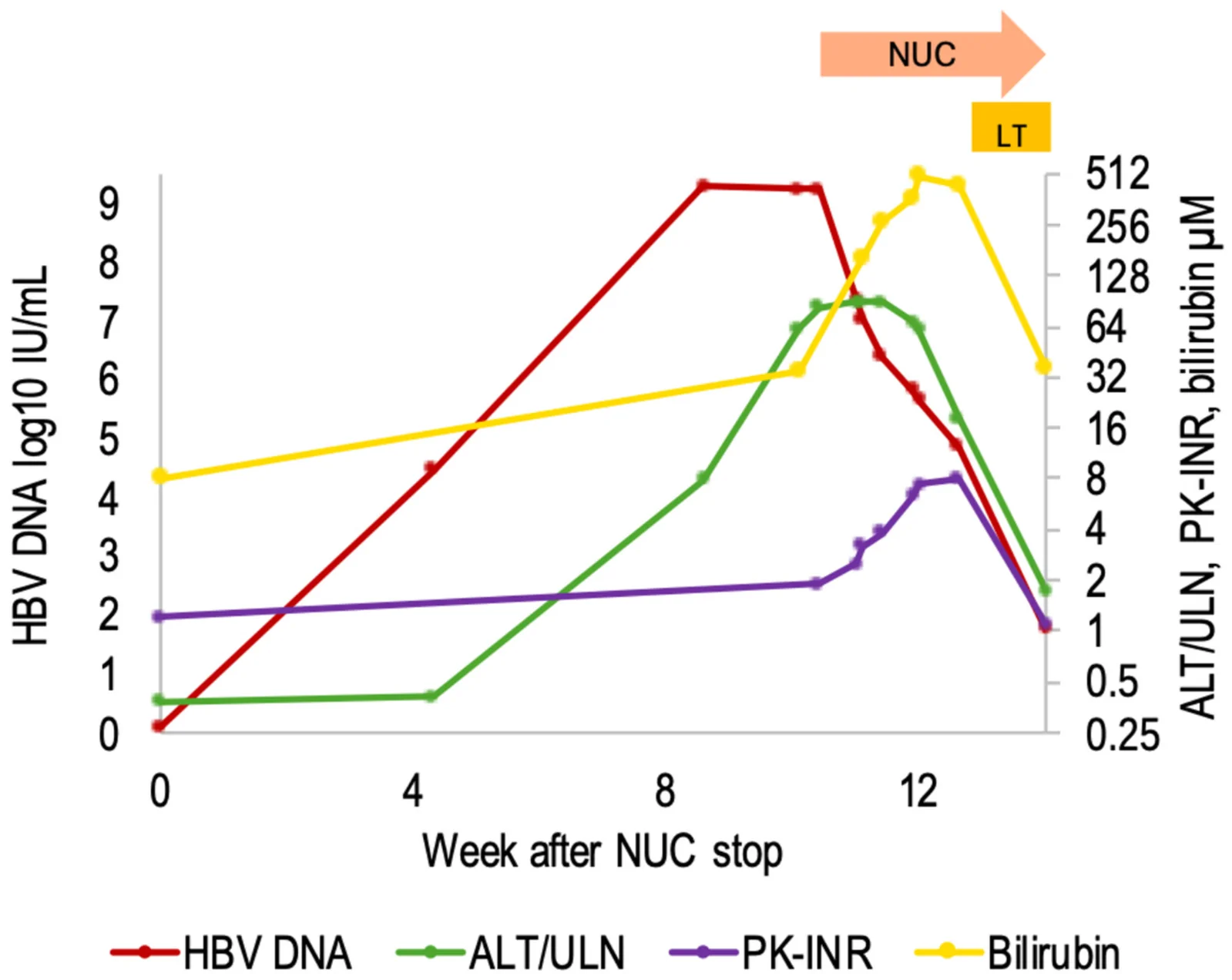
Virological and biochemical development in patient s5 who developed fulminant hepatitis requiring liver transplantation.

One patient (s5) developed fulminant hepatitis requiring liver transplantation. He had received NUC therapy for 15 years; liver stiffness before treatment cessation was 6.2 kPa (F0–F1). Four weeks after stopping NUC, HBV DNA was 4.48 log_10_ IU/mL and ALT/ULN 0.41; 8.6 weeks after stopping NUC HBV DNA had increased to 9.30 log_10_ IU/mL and ALT/ULN to 7.9 (Figure 3). This HBV DNA result was delayed due to technical reasons, and NUC therapy was not restarted until 10.4 weeks after discontinuation, when HBV DNA remained above 9.0 log_10_ IU/mL, ALT/ULN had increased to 92, bilirubin to 35 µM and PK-INR to 1.9. HBV DNA levels then declined rapidly, but ALT increased further. When PK-INR reached 7.3 and bilirubin 494 µM at 12 weeks after NUC discontinuation, liver transplantation was indicated and performed 4 days later.

The patient recovered without complications and remains well 1.5 years after transplantation. As shown in Supplementary Table S3, this patient differed from other male patients older than 50 years and infected with HBV genotype D mainly by having a higher HBcrAg level before stopping NUC therapy.

Another patient (i6) had an initial mild flare at week 8, and a second, late severe flare after week 30, with HBV DNA rising to 8.33 log_10_ IU/mL, ALT 50 × ULN and PK-INR 1.6. These levels rapidly declined when NUC therapy was restarted.

### 3.6. Insufficient Control

Five patients (20%) presented poor or transient reductions in HBV DNA after HBV DNA peaks between 4.94 and 7.23 log_10_ IU/mL and relatively mild ALT elevations (Figure 1D–F, Supplementary Figure S5), indicating insufficient immunological control. Because of persistent HBV DNA levels above 3.3 log_10_ IU/mL and ALT elevations, NUC was restarted 20–96 weeks (median 60 weeks) after discontinuation when HBV DNA levels ranged from 4.94 to 6.99 log_10_ IU/mL (median 5.77 log_10_ IU/mL).

### 3.7. Borderline Control

Three patients (12%) presented moderate reactivations followed by HBV DNA levels persistently above 3.3 log_10_ IU/mL (Figure 1G–I, Supplementary Figure S4). Since ALT levels remained within the normal range, NUC therapy was not restarted.

### 3.8. Virological Biomarker Associations

Most patients presented a distinct HBV DNA peak, which typically preceded the ALT peak by 1–4 weeks. In the control group, which continued NUC therapy, levels of HBV DNA, HBsAg and ALT were largely unchanged during follow-up (Figure 1P–R, Supplementary Figure S7).

**Figure 4:**
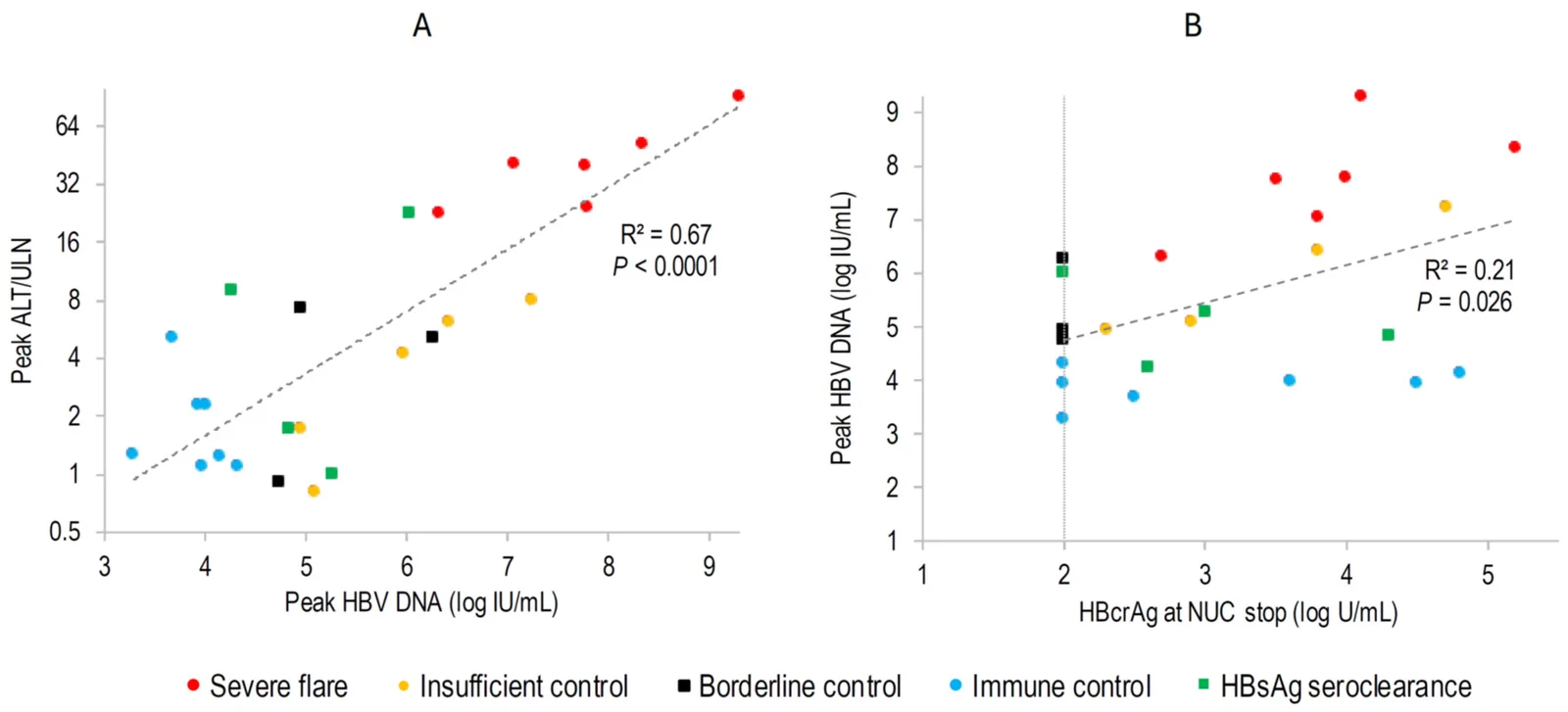
Correlations between (**A**) peak HBV DNA and peak ALT during reactivation after stopping NUC therapy, (**B**) HBcrAg at NUC stop and peak HBV during reactivation. Samples with undetectable HBcrAg are shown as 2.0 log U/mL (limit of detection, dotted vertical line). Dots are color-coded to show outcome group.

As shown in Figure 4A, peak HBV DNA levels correlated strongly with peak ALT (*p* < 0.0001). Patients with severe flares had high peaks for both variables, whereas patients in the immune control group clustered at low levels of both HBV DNA and ALT. Peak HBV DNA levels correlated with the HBcrAg levels at NUC discontinuation (Figure 4B, *p* = 0.026), but not with HBV DNA levels prior to starting NUC therapy, which did not differ significantly between the outcome groups (Figure 2A).

**Figure 5:**
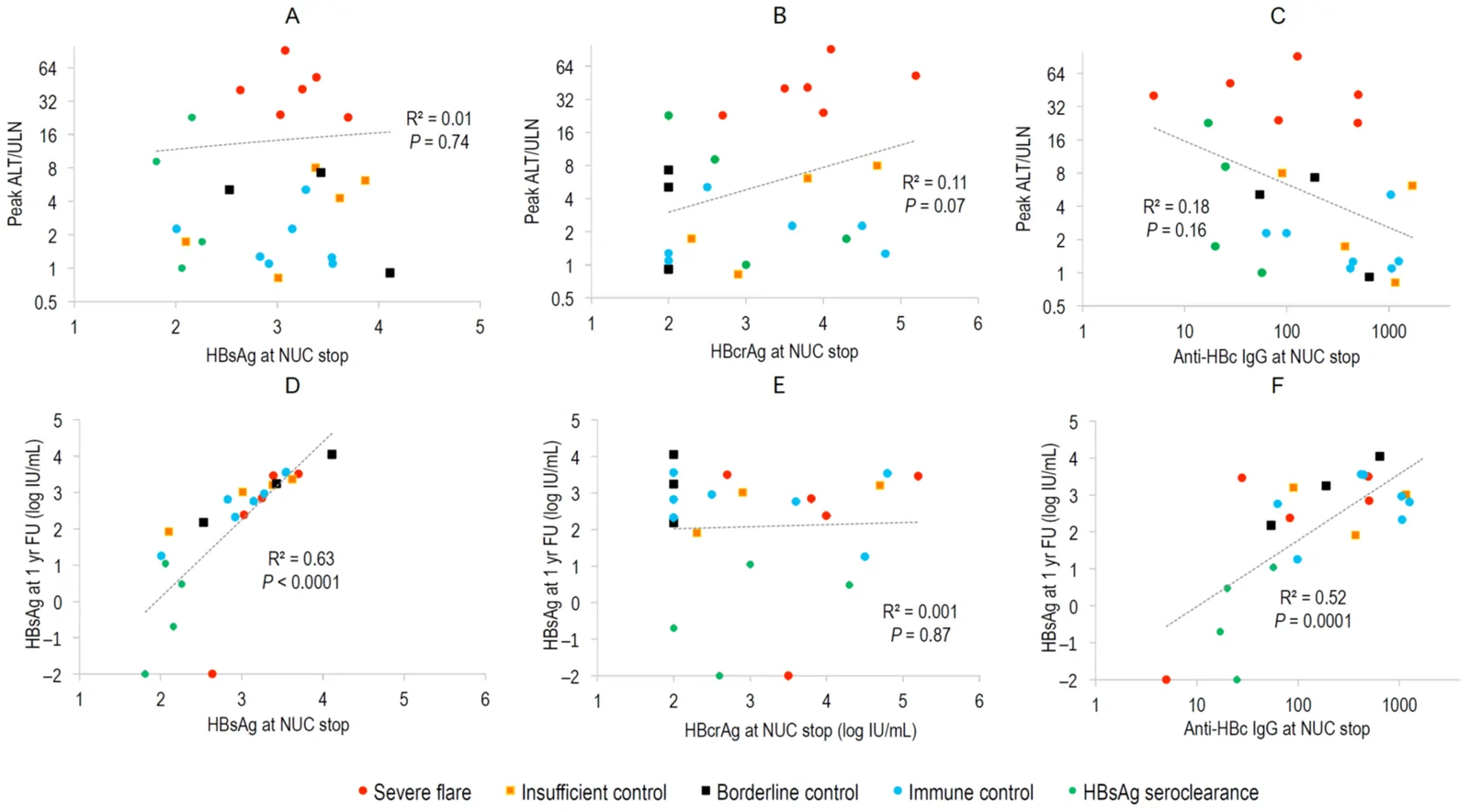
Correlations between peak ALT and (**A**) HBsAg, (**B**) HBcrAg and (**C**) anti-HBc IgG at NUC discontinuation, and between HBsAg level 1 year after NUC discontinuation and (**D**) HBsAg, (**E**) HBcrAg and (**F**) anti-HBc IgG at NUC discontinuation. The dots are colored to show outcome group. Regression lines are based on all values in each plot. Undetectable HBsAg is shown as –2 log IU/mL. Undetectable HBcrAg is shown as 2 log IU/mL (limit of detection).

As shown in Figure 5D, HBsAg at the time of NUC discontinuation generally remained at a similar level during follow-up, except in a few patients with low baseline levels. The median HBsAg level at treatment cessation was significantly lower in the spontaneous HBsAg seroclearance group than in the other groups (Figure 2B). There was no correlation between HBsAg level at NUC discontinuation and peak ALT level (Figure 5A).

HBcrAg was detectable at treatment discontinuation in all patients who developed severe flares, with a median of 3.9 log U/mL, as compared with 2.6 log U/mL in those with other outcomes (*p* = 0.046, Figure 2C). However, no HBcrAg cut-off distinguished those with severe flare from other groups. HBcrAg at treatment discontinuation showed a weak correlation with peak ALT levels (Figure 5B, *p* = 0.07) and was not associated with HBsAg seroclearance (Figure 5E).

The median anti-HBc IgG level at discontinuation of NUC therapy was 105 IU/mL in patients who developed severe flare as compared with 279 IU/mL in those who did not (*p* = 0.092). Four of six patients (66%) with severe flare had a baseline anti-HBc IgG level below the proposed cut-off of 400 IU/mL compared with 10 out of 18 (56%) without severe flare (*p* = 0.69). No significant correlation was found between anti-HBc IgG levels and peak ALT levels (Figure 5C), nor with peak HBV DNA.

Of note, low anti-HBc IgG was associated with HBsAg seroclearance (Figure 2D): Median anti-HBc IgG levels were 22 vs. 394 IU/mL (*p* = 0.0005) for patients with or without the spontaneous HBsAg seroclearance outcome.

## 4. Discussion

Despite the relatively small sample size in this prospective study, three clinically important outcomes could be distinguished and characterized: immune control, HBsAg seroclearance, and severe flare.

Immune control was achieved in seven patients (28%). A similar outcome has been observed after 96 weeks in 27–39% in studies with different proportions of Caucasian and Asian patients, then termed sustained remission, sustained response, or partial cure [4,19,20,21]. This outcome appears to represent an underappreciated benefit of stopping NUC therapy, as attention has largely focused on HBsAg loss as primary endpoint [4,5,7,9,11]. In our study, the patients with the immune control outcome had a marked and sustained reduction in HBV DNA levels, with an average of more than 4 log_10_ IU/mL reduction compared with prior to NUC therapy. No baseline virological marker reliably predicted the immune control outcome. Its most distinctive feature was a limited early viral reactivation, with HBV DNA peaks not higher than 4.31 log_10_ IU/mL and minimal or mild ALT elevations. Although continued follow-up remains warranted, late flares may be uncommon in this group and less intensive long-term monitoring might be sufficient [22].

The rate of HBsAg seroclearance in our study was in line with previous reports in HBeAg-negative, predominantly Caucasian patients [4,5,9,11,23], despite the fact that nine patients (36%) were of East Asian origin and infected with genotype B or C, origins and genotypes generally associated with lower rates of HBsAg loss. Five of twenty-five patients (20%) presented an HBsAg seroclearance pattern, including one who cleared HBsAg after restarting NUC therapy because of a severe flare. The four patients with the spontaneous HBsAg seroclearance outcome had received NUC therapy for longer than the other patients and, in agreement with previous studies [6,11], had lower HBsAg levels at the time of discontinuation.

All five patients in the HBsAg seroclearance group experienced viral reactivations. In two of them, the reactivation was limited and accompanied by a mild biochemical flare. In the other three, reactivation was more pronounced and associated with marked ALT elevations, and these patients also showed a more rapid decline in HBsAg, suggesting that in some patients, a strong cytolytic immune response may be essential for HBsAg clearance [7,24,25]. Such a response might be required to eliminate a sufficient number of hepatocytes containing integrated HBV DNA, which are believed to be a major source of circulating HBsAg in many patients with chronic hepatitis B [26,27].

An additional and unexpected finding was that HBsAg seroclearance was associated with lower anti-HBc IgG levels. This, to our knowledge, has not been reported before, but low anti-HBc IgG levels have been associated with severe flare [14,28].

Severe flares requiring re-initiation of NUC therapy occurred in 6 of 25 patients (24%). One of these patients developed fulminant hepatitis requiring liver transplantation, resembling a case reported previously [29]. In our patient, the increase in HBV DNA four weeks after NUC discontinuation was not exceptional but then continued in a unique manner to a level exceeding 9 log_10_ IU/mL and with pronounced ALT elevation. Despite re-initiation of NUC therapy, the inflammatory response progressed to liver failure, and transplantation was performed 12.4 weeks after NUC therapy was stopped.

This case illustrates that the severity of the biochemical flare is closely linked to the magnitude of the preceding viral rebound, indicating that flare severity depends largely on the extent of intrahepatic viral spread by the time a cytolytic immune response is mounted. In the patient who required transplantation, the extremely high HBV DNA level suggests that infection had spread to a majority of the hepatocytes. In line with recent reports [12,13,14,15], this patient had a detectable and relatively high level of HBcrAg when NUC therapy was discontinued.

Another patient developed a severe flare as late as week 31 after NUC discontinuation, with HBV DNA levels reaching 8.33 log10 IU/mL and ALT rising to 50 times the upper limit of normal, together with an increase in PK-INR to 1.6. This case demonstrates that severe flares can present more than 6 months after discontinuation of tenofovir therapy and underlines the need of a long and close surveillance, at least for patients with an enhanced risk of developing a severe flare.

The optimal monitoring interval remains uncertain and may need to be individualized based on predictive factors and the early post-discontinuation virological pattern. Moreover, safety depends not only on visit frequency but also on rapid HBV DNA turnaround and immediate clinical action. Our data suggest that monthly sampling may be adequate initially, but that closer follow-up should be considered once HBV DNA levels increase above 4 log IU/mL. The optimal threshold for restarting NUC therapy cannot be determined from this small study, but our findings support guidelines stating that levels exceeding 5.0 log_10_ IU/mL should prompt urgent reassessment and consideration of immediate retreatment [30].

Our findings, together with previous reports, also have implications for the decision to initiate long-term NUC therapy in the first place. When therapy can be stopped only with difficulty, intensive monitoring, and non-negligible risk, the expected long-term benefit of starting therapy should be weighed carefully against this risk and discussed with the patient, not least in patients with a less strong indication for treatment. Fortunately, the risk of severe flare in patients with low pretreatment levels of HBV DNA seems to be lower [31].

In line with previous reports, all cases with severe flare had detectable HBcrAg, supporting that this marker is clinically useful to identify patients at risk of severe flares [13,14,15]. In our cohort, exclusion of patients with HBcrAg levels above 3.0 log_10_ U/mL from treatment discontinuation would have prevented all but one severe flare. However, this approach would also have excluded one patient who achieved HBsAg loss and two who achieved immune control. Another potential marker for predicting severe flare is anti-HBc IgG [14,15,16]. In our cohort, the proposed cut-off of 400 IU/mL identified only four of the six patients who developed severe flare, while also excluding all four patients with a spontaneous HBsAg seroclearance pattern. These observations suggest that these predictive markers may be more useful for risk stratification and tailoring post-discontinuation monitoring than for categorically ruling out treatment cessation.

The 2025 EASL guidelines state that NUC discontinuation should be restricted to patients with low HBsAg levels, defined as <100 IU/mL for Asians and <1000 IU/mL for non-Asians. In our study, NUC would have been discontinued in only two patients if persons from the Middle East were considered as ‘Asians’ (one achieving HBsAg loss, the other immune control), and in six patients (three achieving HBsAg seroclerance, two immune control, one borderline control) if the <100 IU/mL cut-off was not applied to patients from the Middle East. With the latter selection, no patient developing severe flare would have been included, but neither would 5 of 10 patients with beneficial outcomes (2/5 in the HBsAg seroclearance group; 3/5 in the immune control group).

In summary, discontinuation of NUC therapy in HBeAg-negative CHB resulted in favorable outcomes in approximately half of the patients, including HBsAg seroclearance or sustained immune control without retreatment. However, 24% developed severe flares, including one case of fulminant hepatitis requiring liver transplantation. Low HBsAg levels were associated with subsequent HBsAg seroclearance, whereas higher HBcrAg levels were associated with severe flare. These findings underline the importance of careful patient selection and intensive monitoring when finite NUC therapy is being considered.

## Figures and Tables

**Table 1:** Baseline characteristics of patients stopping and controls continuing NUC therapy.

	Patients Stopping NUC	Controls
	*n* = 25	*n* = 6
Male/female	14/11	3/3
Age at NUC-stop ^a^	54.9 (43.0–72.5)	51.0 (31.4–73.5)
Duration of NUC therapy ^a^	10 (4–20)	10 (7–18)
Pre-NUC HBV DNA ^b^	5.94 (3.28–8.10)	7.26 (5.92–7.9)
HBV genotype A/B/C/D/E	1/4/5/16/1	1/2/0/3/0
Geographic origin (SE/ME/EA/CSA/A) ^b^	5/8/9/2/1	1/2/2/0/0
HBsAg at NUC-stop ^b^	3.08 (1.81–4.11)	2.70 (2.12–3.71)
<2 log_10_ IU/mL	1	
2–3 log_10_ IU/mL	9	4
3–4 log_10_ IU/mL	14	2
>4 log_10_ IU/mL	1	
HBcrAg at NUC-stop ^b^	2.95 (<2–5.2)	3.20 (<2–4.2)
Detected	17	5
Not detected	7	1
Anti-HBc IgG at NUC-stop ^c^	158 (5–1694)	79 (28–403)
Liver stiffness ^d^	5.5 (3.8–10.7)	5.0 (3.2–5.6)

^a^ Median (range), ^b^ Median log_10_ U/mL (range); missing in one patient, ^c^ Median log_10_ IU/mL (range), ^d^ Median kPa, by FibroScan.

**Table 2:** Characteristics and liver damage markers in patients who stopped NUC therapy.

	HBsAg Seroclearance (Spontaneous)	Immune Control	Borderline Control	Insufficient Control	Severe Flare	*p* Value ^d^
	*n* = 4	*n* = 7	*n* = 3	*n* = 5	*n* = 6	
Age (years) at NUC stop ^a^	62.2 (55–74)	43.1 (31–65)	59.6 (38–61)	52.9 (47–55)	48.3 (43–59)	0.15
Duration of NUC therapy	14 12–20	10 4–16	10 8–12	5 5–10	11 4–15	0.042
Geographic origin (SE/ME/EA/CSA/A) ^b^	1/2/1/0/0	1/3/2/1/0	0/2/0/0/1	1/0/4/0/0	2/1/2/1/0	0.39
HBV genotype (A/B/C/D/E)	0/0/1/3/0	0/1/1/6/0	0/0/0/2/1	1/2/2/1/0	0/1/1/4/0	0.57
At baseline (NUC stop)						
Albumin (g/L) ^a^	42 (37–42)	42 (38–45)	46 (35–51)	42 (40–45)	42 (39–46)	0.81
Platelets (count/µL) ^a^	126 (115–210)	223 (165–361)	207 (193–235)	224 (187–275)	195 (157–277)	0.17
Liver stiffness (kPa) ^a,c^	5.6 (4.6–7.2)	4.5 (3.8–10.7)	5.2 (4.3–6.3)	6.5 (5.4–10.4)	5.6 (4.1–8.0)	0.32
During follow-up after NUC stop						
Peak HBV DNA (log_10_ IU/mL)	5.04 4.26–6.02)	3.96 (3.29–4.31)	4.94 (4.74–6.26)	5.96 (4.94–7.23)	7.77 (6.32–9.30)	0.0005
Peak ALT/ULN ^a^	5.4 1–23	1.3 1.1–5.1	5.1 0.9–7.3	4.3 0.8–8.0	40 23–91	0.0077
Peak PK-INR ^a^	1.2 (1.1–1.2)	1.1 (1–1.4)	1.2 (1.1–1.2)	1.1 (1–1.3)	1.4 (1.2–8)	0.11
Peak bilirubin (mM) ^a^	18 (14–48)	10 (6.9–28)	20 (8–24)	13 (7.8–18)	19 (12–494)	0.17

^a^ Median (range), ^b^ SE, South Europe; ME, Middle East; EA, East Asia; CSA; Central–Southern Asia; A, Africa. ^c^ by FibroScan elastography. ^d^ By Kruskal–Wallis test.

## Data Availability

Due to confidentiality aspects, detailed individual data cannot be shared, but results at group level are available on request to the corresponding author.

## Supplementary Materials

The following supporting information can be downloaded at: https://www.mdpi.com/article/10.3390/v18091026/s1, Table S1: Consort checklist; Table S2: Virological parameters in patients with different outcomes after stopping NUC treatment; Table S3: Comparisons of potential predictors of severe flare between the case with fulminant hepatitis requiring transplantation and other male patients with age above 50 years, treated with tenofovir and infected with HBV genotype D; Figure S1. Consort flow chart; Figure S2: Spontaneous HBsAg seroclearance patterns; Figure S3: Immune control cases; Figure S4: Borderline control cases; Figure S5. Insufficient control cases; Figure S6. Severe flare cases; Figure S7. Control group cases; Figure S8. HBV DNA levels in the immune control group before starting and after stopping NUC therapy. Ref [32] is cited in Supplementary File.
