# Supplementary material for: Immune Control, HBsAg Loss or Flare Requiring Liver Transplant—Diverse Outcomes After Stopping NUC Therapy
Source: Viruses. 2026 Sep 15;18(9):1026. doi: 10.3390/v18091026 (PMC13611910; doi:10.3390/v18091026)
Supplement: Supplementary file 1 [file viruses-18-01026-s001.zip › viruses-4501641-supplementary.pdf]

Table S1. Consort checklist*

Section / Topic	No	SPIRIT 2025 checklist item description
**Administrative information**		
Title and structured summary	1a	Discontinuation of Antiviral Therapy as a Strategy to Cure Hepatitis B (STOP-B). Prospective study of the effect of stopping nucleoside analogue treatment of HBeAg-negative chronic hepatitis B.
	1b	Methods: Patients without liver cirrhosis who had been treated at with nucleoside analogue for > 3 years at an infectious disease clinic were offered to participate in a study that randomised patients to stop or continue treatment in a 3:1 fashion.
Roles and responsibilities	3a	Principal investigator: Magnus Lindh, University of Gothenburg, was responsible for design of the study, data collection and data analysis.
**Open science**		
Trial registration	4	Clinicaltrial.com. NCT05328427.
Protocol and statistical analysis plan	5	Protocol can be obtained at request to Magnus Lindh, Guldhedsgatan 10 B 413 46 Gothenburg, Sweden
Data sharing	6	Data at group level and to some extent at individual level can be obtained at request.
Funding and conflicts of interest	7a	Funding was obtained from the Swedish government and from the Swedish Cancer foundation to principal investigator.
	7b	The principal investigator (Magnus Lindh) has no conflict of interest.
Dissemination policy	8	The results will be published in peer-reviewed scientific journals.
**Introduction**		
Background and rationale	9a	Previous studies have reported that stopping NUC treatment may increase the chance of HBsAg loss. The 2017 EASL CPG recommended that stopping NUC could be used as a strategy for obtaining HBsAg loss but preferably within a clinical study setting.
Objectives	10	To study the rate of (i) HBsAg loss, (ii) achievement of persistently low viral replication, (iii) severe flare. (iv) Identification of markers associated with these outcomes.
**Methods: Patient and public involvement, trial design**		
Patient and public involvement	11	Public involvement was not planned.
Trial design	12	Prospective randomized trial.
**Methods: Participants, interventions, and outcomes**		
Trial setting	13	Three infectious disease clinics in western Sweden (uptake population approximately 1 million).
Eligibility criteria	14a	Chronic HBeAg-negative hepatitis B treated with a nucleoside analogue for > 3 years. Age ≥ 18 years.
	14b	No cirrhosis, absence of anti-HCV, anti-HDV, anti-HIV.
Intervention and comparator	15a	Intervention: Stopping NUC therapy. Comparator: Continuing NUC therapy.
Outcomes	16	HBsAg clearance.<br>HBV DNA < 3.3 log IU/mL and ALT ≤ ULN.<br>Severe flare (ALT/ULN > 10).
Harms	17	Severe flare (as indicated by ALT/ULN > 10 in combination with abnormal markers for liver failure, mainly PK-INR>1.4)
Participant timeline	18	Inclusion between December 2022 until December 2024.

Sample size	19	A sample size of 80 was calculated to be sufficient to identify a greater likelihood of HBsAg loss and/or achievement of normal ALT and low HBV DNA as compared with controls with a statistical significance.
Recruitment	20	Patients on NUC therapy were informed about the study when they attended routine visits (scheduled twice per year). If they were interested in participating, they were informed further at the following visit to their doctor.
**Methods: Assignment of interventions**		
Randomization:	21	Randomization by computer algorithm. 3:1 ratio.
Allocation concealment mechanism	22	Obtained from central computer.
Implementation	23	Randomization obtained at the time of inclusion.
Blinding	24a	No.
**Methods: Data collection, management, and analysis**		
Data collection methods	25a	Biochemical data obtained from electronic CRF. Virological data obtained from microbiology LIS (laboratory information system). Additional data from patient record.
Data management	26	Data (results for each patient, with patient ID coded) were entered by a study nurse (with personal login) in an electronic CRF.
Statistical methods	27a	Comparison of outcomes: Mann-Whitney U test, Fisher's exact test, Kruskal–Wallis test, logistic regression, as appropriate (decision by statistician involved in the study).
**Methods: Monitoring**		
Data monitoring committee	28a	No DMC.
Trial monitoring	29	By study nurse at each site and by principal investigator.
**Ethics**		
Research ethics approval	30	Approval by the Swedish National Ethical Review Authority (Dnr 2019-04563)
Consent or assent	32a	Written informed content was obtained from each participant.
Confidentiality	33	Coded study ID for each patient. All information about the patients was recorded in study data base under this code.
Ancillary and post-trial care	34	No provisions. Standard patient insurance valid.

Citation: Chan A-W, Boutron I, Hopewell S, Moher D, Schulz KF, et al. SPIRIT 2025 statement: updated guideline for protocols of randomised trials. BMJ 2025;389:e081477. https://dx.doi.org/10.1136/bmj-2024-081477

Table S2. Virological parameters in patients with different outcomes after stopping NUC treatment

	Severe flare	Insufficient control	Borderline control	Immune control	Spontaneous HBsAg seroclearance	P value
	n=6	n=5	n=3	n=7	n=4	
Pre-NUC HBV DNA[a]	6.66	5.60	5.73	6.26	6.82	NS
	5.19–7.33	5.08–7.81	5.67–5.8	0.45–8.10	5.12–7.37	
HBsAg at NUC stop[a]	3.16	3.34	3.43	3.21	2.11	0.0005[c]
	2.64–3.7	2.1–3.87	2.53–4.11	2.01–3.55	1.81–2.26	
HBcrAg at NUC stop[b]	3.9	3.0	<2.0	2.4	2.8	0.046[d]
	2.7–5.2	2.3–4.7	<2–<2	<2–4.8	<2–4.3	
Anti-HBc at NUC stop[a]	105	1161	188	746	22.5	0.0005[c]
	1–501	90–3240	54–641	63–1606	17–57	
HBsAg at 1 yr FU[b]	2.85	3.13	3.65	2.88	-0.11	<0.0001[c]
	(-2–3.5)	(1.91–3.36)	(3.24–4.05)	(1.26–3.56)	(-2–1.04)	
HBsAg at 2 yrs FU[b]	2.54	3.17	3.24	2.74	-2	<0.0001[c]
	(-3–3.34)	(1.71–3.84)	(2.12–4)	(1.26–3.52)	(-3–-0.05)	

[a] $\log_{10}$ IU/mL. [b] $\log_{10}$ U/mL. [c] Spontaneous HBsAg seroclearance vs. other. [d] Severe flare vs. other.

Table S3. Comparisons of potential predictors of severe flare between the case with fulminant hepatitis requiring transplantation and other male patients with age above 50 years, treated with tenofovir and infected with HBV genotype D.

ID	Outcome after NUC stop	Age[a]	HBV DNA preNUC[b]	NUC duration[a]	Findings prior to NUC stop					
					Liver stiffness[c]	HBsAg[b]	HBcrAg[d]	Anti-HBc IgG[b]	Albumin[e]	Platelets[f]
s3	Fulminant hepatitis (transplantation)	59	6.07	15	6.20	3.08	4.1	127	46	157
n11	Severe flare	51	5.19	4	5.20	3.25	3.8	501	39	202
i17	HBsAg sero-clearance	73	6.87	20	7.20	1.81	2.6	25	37	124
i8	HBsAg sero-clearance	64	6.76	12	4.60	2.16	<2	17	42	115
i14	HBsAg sero-clearance	61	7.37	14	5.80	2.06	3	57	41	210
n4	Immune control	62	6.26	11	4.30	2.92	<2	1064	44	361
n1	Borderline	61	5.67	12	5.20	2.53	<2	54	51	235
i13	Borderline	60	5.73	10	4.30	3.43	<2	188	46	207

[a] Years, [b] $\log_{10}$ IU/mL, [c] kPa, [d] U/mL, [e] g/L, [f] count/µL

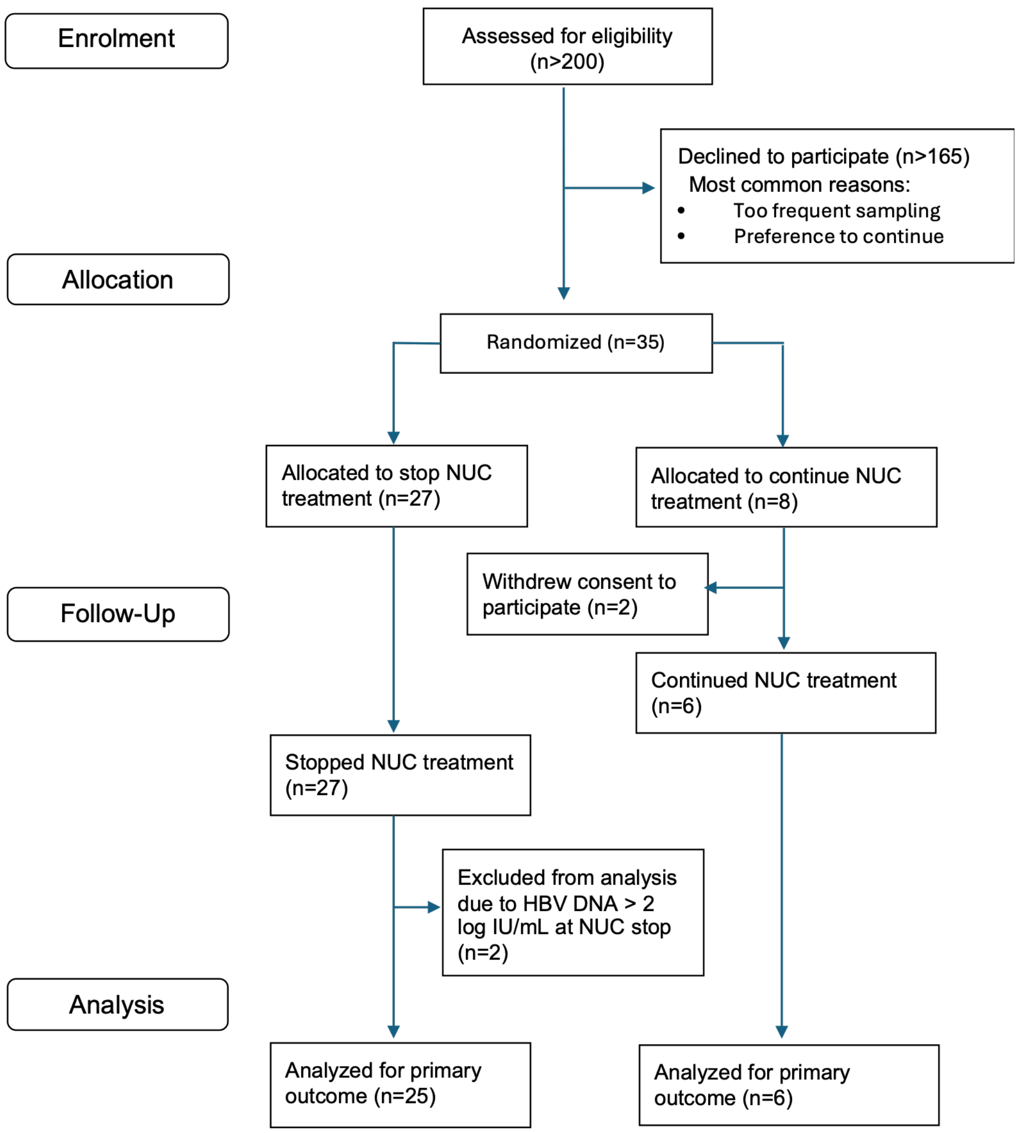

Figure S1. Consort flow chart.

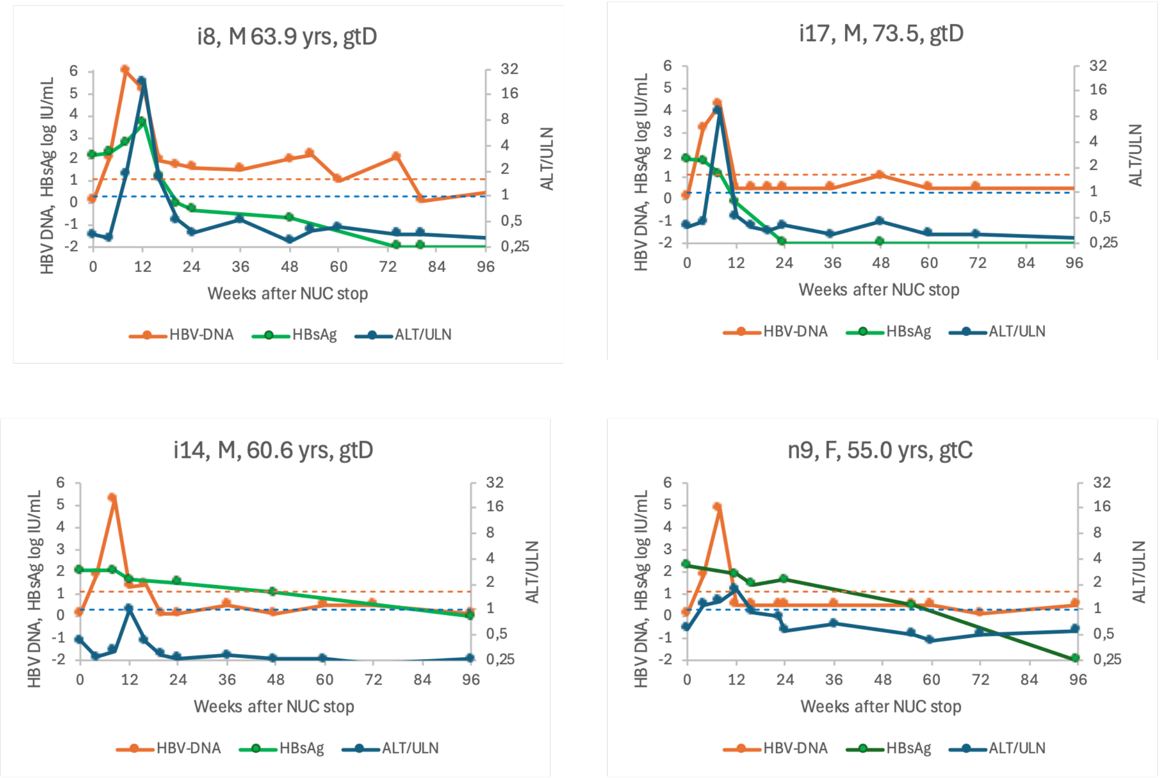

Figure S2. Spontaneous HBsAg seroclearance pattern cases. Plots showing virological and biochemical reactivations after NUC discontinuation in individual patients with a spontaneous HBsAg seroclearance pattern. All patients had received tenofovir.

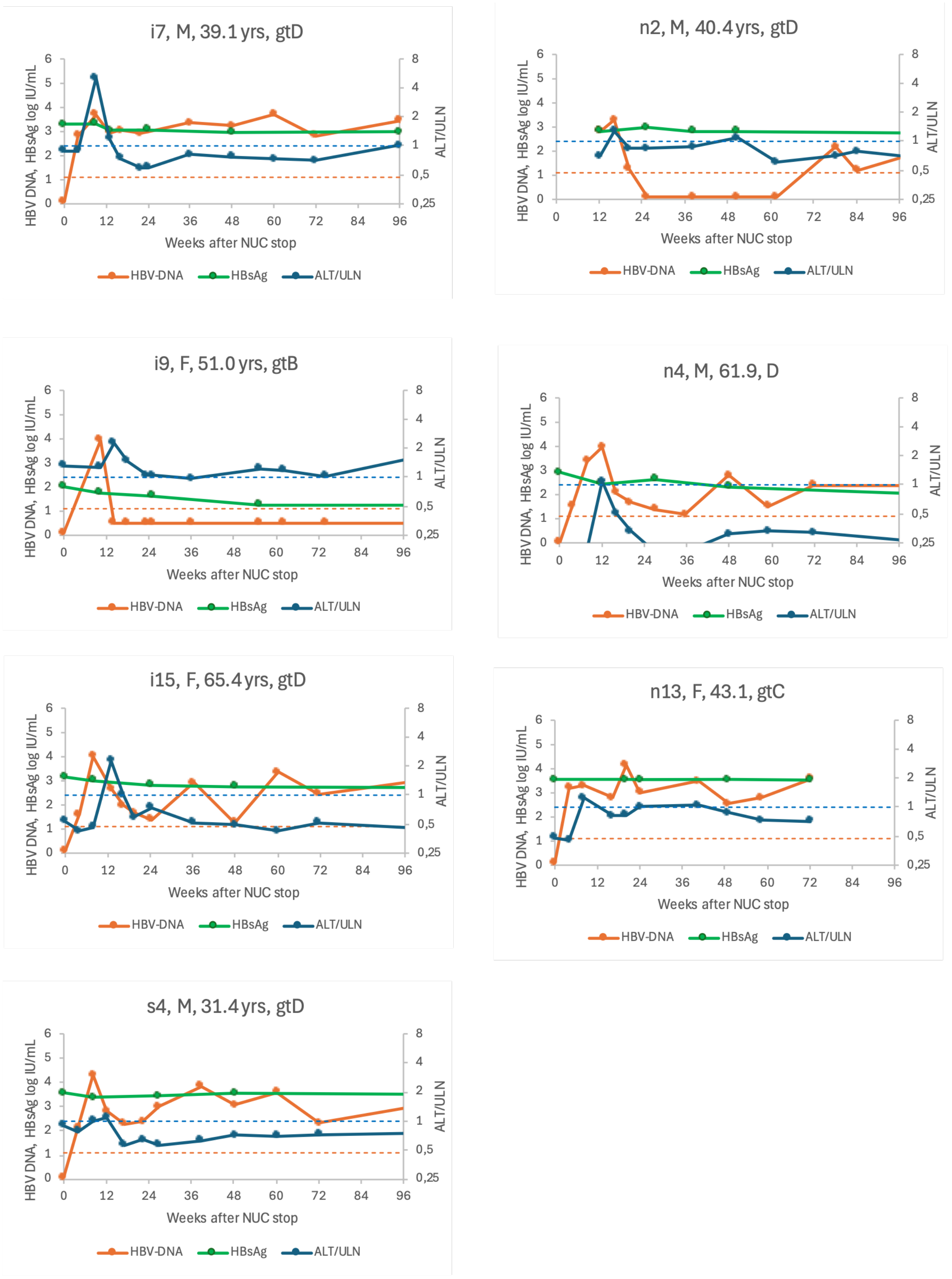

Figure S3. Immune control cases. Plots showing virological and biochemical reactivations after NUC discontinuation in individual patients with immune control (normal ALT and HBV DNA < 3.3 log IU/ml). All patients had received tenofovir.

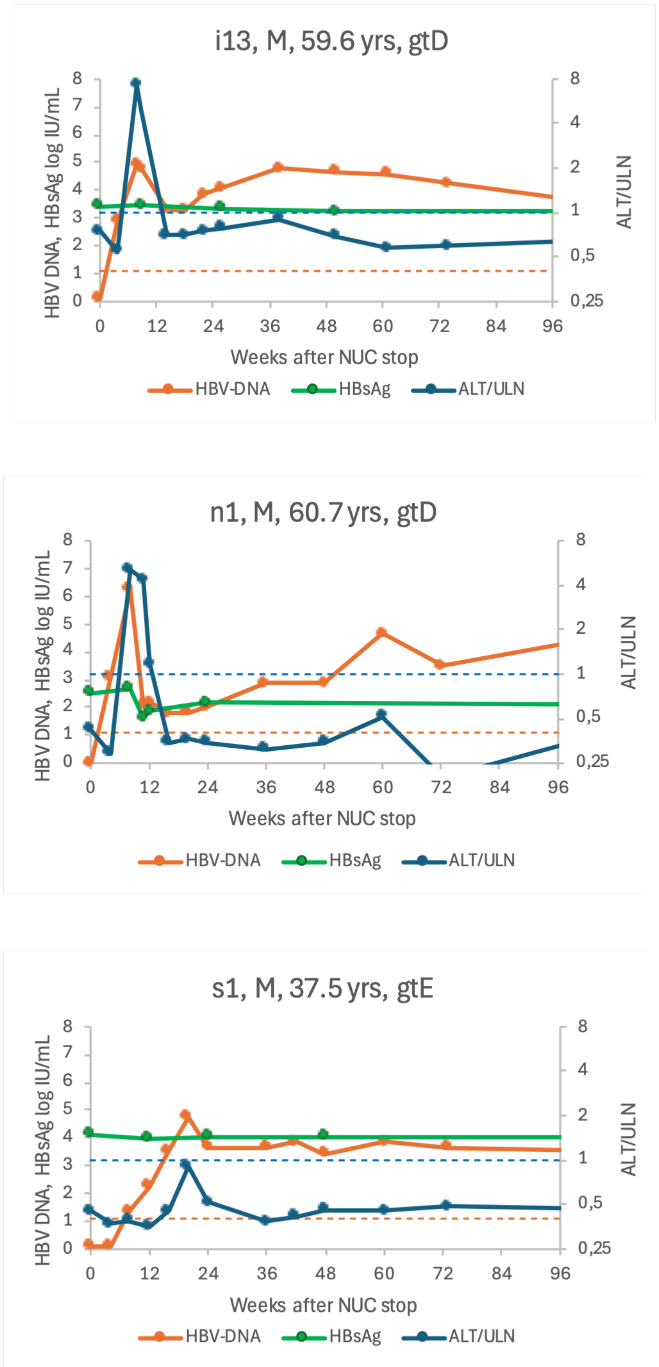

Figure S4. Borderline control cases. Plots showing virological and biochemical reactivations after NUC discontinuation in individual patients with borderline control (normal ALT and HBV DNA > 3.3 log IU/mL). Patient s1 had received entecavir, the other patients tenofovir.

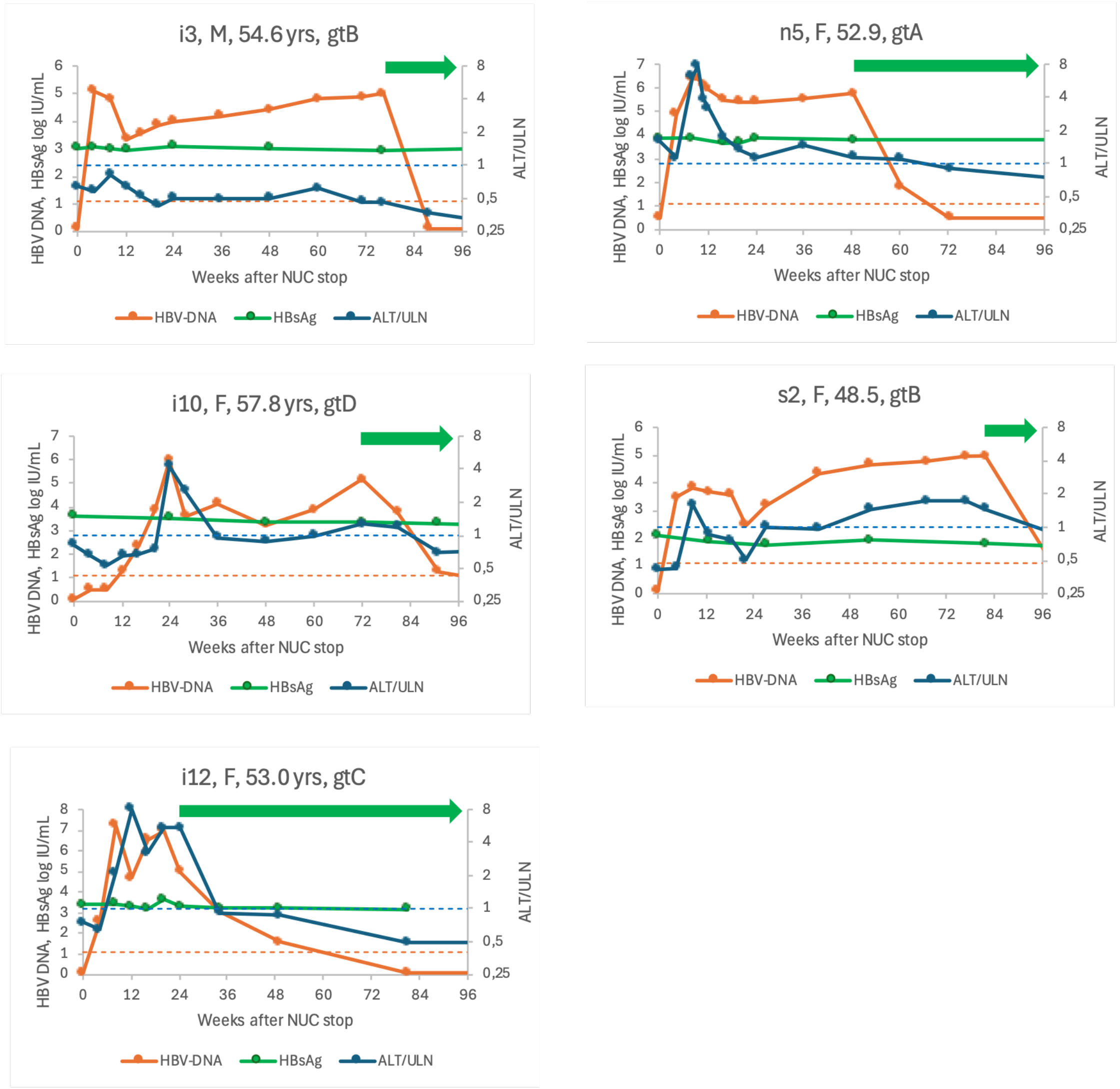

Figure S5. Insufficient control cases. Plots showing virological and biochemical reactivations after NUC discontinuation in individual patients with insufficient control. Patient i10 had received entecavir, the remaining patients tenofovir. Green arrows represent restarted NUC treatment. The dotted blue line shows upper limit of normal for ALT, the dotted orange line limit of detection for HBV DNA.

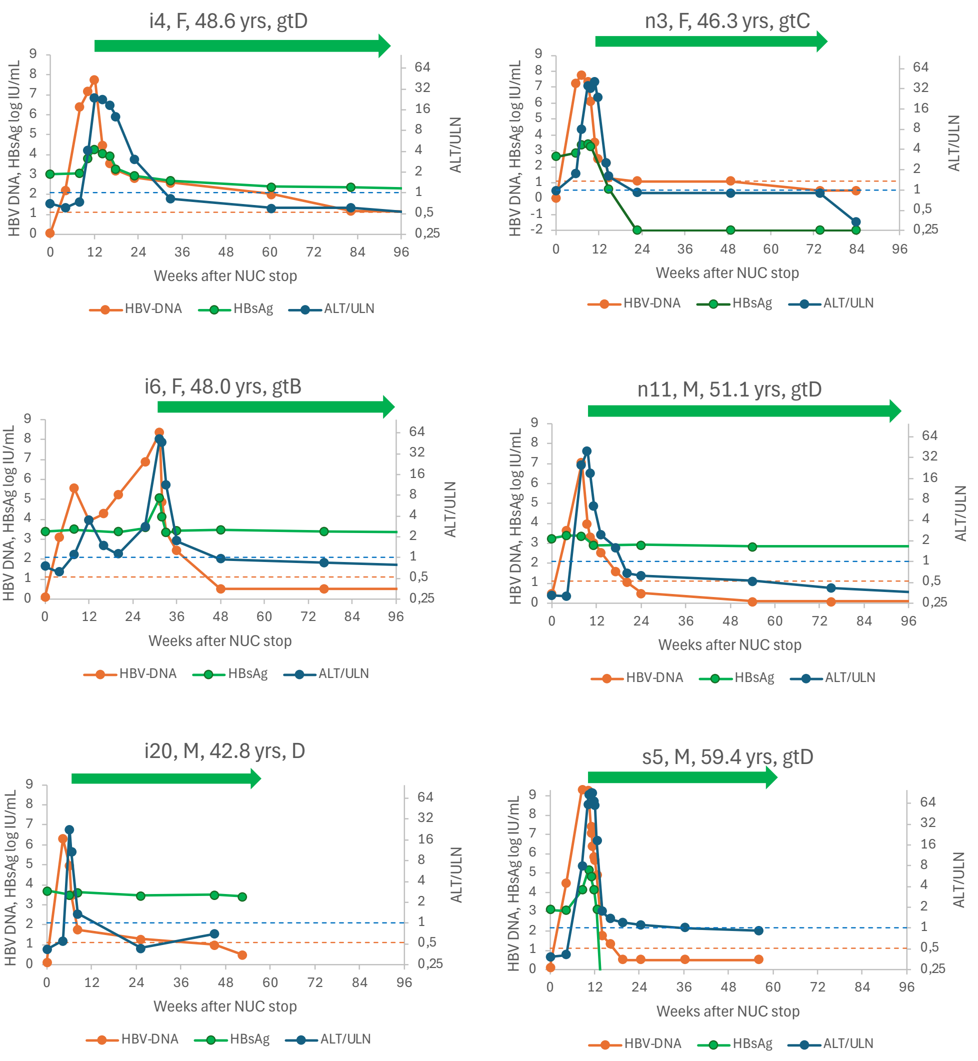

Figure S6. Severe flare cases. Plots showing virological and biochemical reactivations after NUC discontinuation in individual patients with severe flare. All patients had received tenofovir. Green arrows represent restarted NUC treatment.

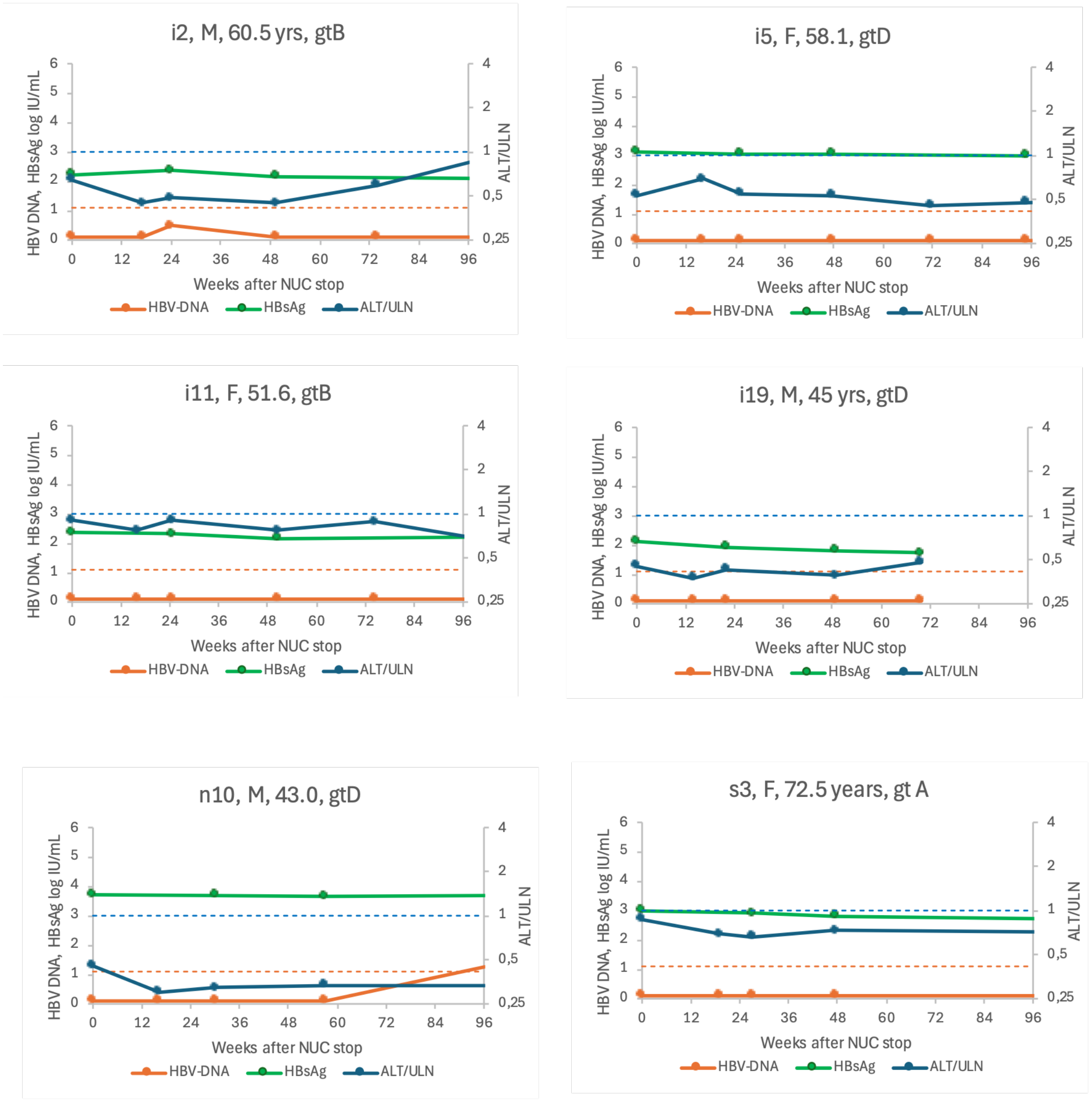

Figure S7. Control group cases. Plots showing virological and biochemical parameters in individual patients who continued NUC treatment (in the control group).

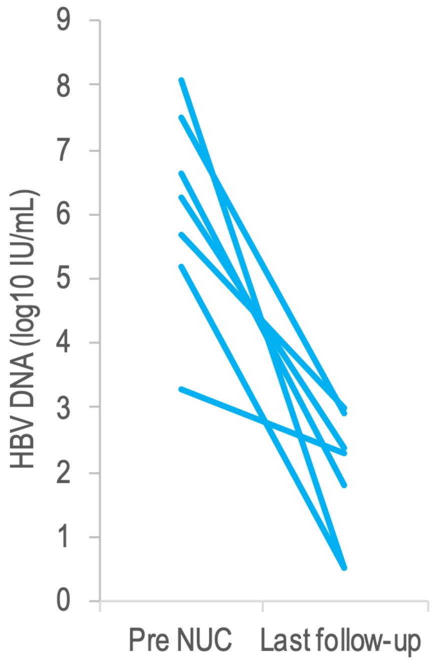

Figure S8. HBV DNA levels in the immune control group before starting and after stopping NUC therapy.
